# Outcomes Following Radiotherapy for Oligoprogressive NSCLC on Immune Checkpoint Inhibitors: A Real-World, Multinational Experience

**DOI:** 10.3390/cancers18010071

**Published:** 2025-12-25

**Authors:** Umair Mahmood, Eleni Josephides, Nicholas Coupe, Daniel Smith, Shahreen Ahmad, Omar Al-Salihi, Sze M. Mak, Meenali Chitnis, Alexandros Georgiou, Daniel Ajzensztejn, Eleni Karapanagiotou, Geoff S. Higgins, Niki Panakis, Jonathan D. Schoenfeld, Michael Skwarski

**Affiliations:** 1 Department of Oncology, Guy’s and St. Thomas’ NHS Foundation Trust, London SE1 9RT, UK; 2 Oxford Cancer and Haematology Centre, Oxford University Hospitals NHS Foundation Trust, Oxford OX3 7LE, UK; 3 Department of Radiology, Guy’s and St. Thomas’ NHS Foundation Trust, London SE1 9RT, UK; 4 Department of Radiation Oncology, Dana-Farber Cancer Institute, Boston, MA 02215, USA

**Keywords:** non-small cell lung carcinoma, oligometastases, radiotherapy, immune checkpoint inhibitors, survival outcomes

## Abstract

**Purpose**: We conducted the largest multinational review to date evaluating outcomes following radiotherapy for non-small cell lung carcinoma (NSCLC) patients with oligoprogressive disease (OPD) on immune checkpoint inhibitors (ICIs). **Methods**: Patients with NSCLC irradiated to ≤5 progressive lesions while receiving ICIs between 2010 and 2023 were identified. We evaluated predictors of local control (LC), progression-free survival (PFS), and overall survival (OS). Patient demographics, disease characteristics, and survival were analyzed using the Wilcoxon test, Kaplan-Meier methods, and uni-/multivariate Cox models. **Results**: Out of 1178 treated patients, 103 eligible ones were included. The median OPD lesion was 1; the most common site was the lung (n = 33). The median LC of irradiated OPD lesions was not reached. Median PFS and OS were 6.90 (5.75–12.91) and 23.46 (17.54–37.16) months, respectively. Patient demographics, tumor pathological factors, number of OPD lesions, cumulative tumor volume, radiation modality, and OPD response to prior ICIs before radiation were not associated with these three outcomes. However, LC was associated with intermediate/high radiation doses (*p* = 0.005) and local response to radiation (*p* = 0.007). Improved PFS was associated with visceral OPD sites following radiation (*p* = 0.01). A favorable OS was associated with intermediate/high radiation doses (*p* = 0.01), local response to radiation (*p* = 0.006), and duration of last ICI before OPD (*p* = 0.03). **Conclusions**: Promising outcomes were observed with ICI and radiation for visceral OPD at intermediate/high doses. Prolonged ICI use before OPD and local response to radiotherapy improved survival. These data can contribute towards guidance of multidisciplinary clinical decision-making for managing OPD in NSCLC patients receiving ICIs.

## Simple Summary

NSCLC patients with oligoprogression on immune checkpoint inhibitors (ICIs) represent an emerging clinical population, but there is limited data to guide clinical decision-making as to the optimal treatment strategy. We aimed to identify clinical and pathological factors in NSCLC that improve stratification of patients most likely to benefit from radiotherapy for oligoprogressive disease with or without ICIs. Because ICIs can offer durable responses with fewer toxicities than other systemic therapies, determining which patients can safely remain on ICIs is clinically valuable. We aimed to identify which patients benefit meaningfully from radiation and which are unlikely to benefit and may require alternative systemic strategies. This study has the potential to refine patient selection, reduce unnecessary radiation exposure, and improve treatment personalization. In addition to informing clinical practice, our data can also guide future trials evaluating radiation for oligoprogressive NSCLC as an alternative to changing systemic therapy or pursuing more invasive local surgery.

## 1. Introduction

Immune checkpoint inhibitors (ICIs) represent a standard therapeutic approach for cancer patients across a spectrum of solid and hematologic malignancies, but clinical responses are variable [1]. Among responders, especially with lung cancer, the emergence of acquired resistance remains a significant challenge and may manifest as oligoprogressive disease (OPD) [2]. This phenomenon complicates clinical decision-making when selecting between localized interventions versus changing systemic treatment.

Local therapy has an increasingly established role in the treatment of oligometastases, supported by randomized trials demonstrating improvements in survival outcomes [3,4,5,6,7]. Within this context, OPD has emerged as a distinct subset of the oligometastatic spectrum, characterized by the progression of only limited metastatic lesions following systemic therapy [8]. However, evidence regarding optimal treatment outcomes for OPD remains limited, posing challenges for clinicians in selecting the most effective management strategies. In some scenarios, switching systemic therapy may be appropriate to address disseminated resistance to systemic therapy, whereas in other cases, localized therapy can potentially ablate isolated therapy-resistant metastases [8]. Radiotherapy, including ablative stereotactic techniques, is increasingly favored for localized progression while maintaining systemic therapy—especially in non-small cell lung cancer (NSCLC), where early studies suggest it can extend disease control beyond six months in select patients [9].

The administration of ICIs is generally associated with a different toxicity profile compared with conventional systemic therapies among NSCLC patients. While ICIs may demonstrate lower overall rates of grade ≥3 adverse events than docetaxel or platinum-based chemotherapy in clinical trials, they are associated with distinct immune-related adverse events, including pneumonitis, colitis, endocrinopathies, and hepatitis, which can be severe, unpredictable, and occasionally life-threatening [10,11,12,13]. Conversely, platinum-based chemotherapy and combination cytotoxic regimens are more frequently associated with high-grade hematologic, gastrointestinal, and neurologic toxicities that can limit treatment duration and tolerability. Thus, although ICIs are often perceived as better tolerated, their toxicity profile requires careful patient selection and monitoring.

The identification of patients developing OPD while on ICIs who would be ideal candidates for local therapy is of particular clinical interest, given the potential for durable clinical benefit and the relatively favorable safety profile of ICIs compared to other systemic agents [14]. Therefore, we performed a real-world evidence analysis of treatment response, patterns of progression, and survival outcomes after ICI and radiotherapy for OPD in NSCLC patients. We also endeavored to identify variables predictive of local control and survival to support more informed decision-making regarding the future application of ICIs and radiotherapy among these patients.”

## 2. Methods

### 2.1. Patient Selection

This retrospective study was approved by the Guy’s Cancer Cohort at the Guy’s and St. Thomas’ NHS Foundation Trust and the ethics committee at Oxford University Hospitals NHS Foundation Trust. Data from the Dana-Farber/Harvard Cancer Center (DF/HCC) cohort was not collected anew, as it involved compilation and re-analysis of previously published data from a pan-cancer study [15]. Adult patients with NSCLC treated from January 2010 to April 2023 were identified (N = 1178). Eligible patients were diagnosed with histologically confirmed NSCLC, with or without metastatic sites, and presented with ≤5 progressive lesions while receiving a single-agent ICI or in combination with other agents, which were subsequently treated with any type of radiotherapy. ICI agents are comprised of Nivolumab, Pembrolizumab, Durvalumab, Atezolizumab, and Ipilimumab. Oligoprogressive lesions were identified using standard clinical imaging methods for all except one patient, who was identified using clinical observation. All identified OPD lesions were radiated. Patients were eligible if all remaining unirradiated disease sites were stable or responsive to ICI. These unirradiated lesions remained unchanged within 3 months of commencing radiation unless the patient developed progressive disease. Patients were excluded if systemic therapy was changed without evidence of disease progression. OPD was defined as involvement of 1–5 lesions, in accordance with consensus recommendations from the ASTRO-ESTRO joint committee [16].

Oligoprogressive lesions were identified according to the RECIST 1.1 criteria as (1) the appearance of new lesions during ICI therapy, (2) progression of previously stable or responding lesions, or (3) continued progression of existing lesions since initiation of ICI.

We abstracted data from patient medical records, including reports regarding next-generation sequencing or programmed death-ligand 1 (PD-L1) expression levels. Data regarding treatment-related adverse events (TRAEs) were also collated. Events were classified either based on the Common Terminology Criteria for Adverse Events v4.0 when available or as severe/non-severe based on physician documentation or if the event necessitated hospital admission.

### 2.2. Outcomes and Statistical Considerations

Outcome measures included local control of irradiated OPD lesions (LC), progression-free survival (PFS; defined as time from the first radiation fraction to first progression at either irradiated or non-irradiated sites), and overall survival (OS).

Assessment of oligoprogressive lesion response adhered to RECIST v1.1 criteria, utilizing comparisons between pre-radiotherapy imaging and serial follow-up imaging performed at approximately three-month intervals. RECIST-based evaluations were retrospectively applied to radiology reports for all OPD lesions or sourced from earlier published analyses for the DF/HCC cohort [15]. For OPD bone lesions (n = 11) in 8 patients, response was determined from physician-reported assessments.

Continuous variables, including age, duration of treatment with the last ICI prior to OPD diagnosis, and cumulative OPD volume, were summarized using median and interquartile ranges (IQR). Categorical variables were summarized using frequency distributions and contingency tables. These included gender, smoking status, PD-L1 expression and mutation status, prior treatment, type of ICI immediately preceding radiotherapy for OPD lesions, best overall response to the last ICI, response of OPD lesions to prior ICI, number of OPD lesions, anatomic site of irradiated OPD lesions, re-irradiation of OPD lesions, radiation modality used for OPD lesions, radiation dose regimen categorized by EQD2 levels, best local response of irradiated OPD lesions, treatment-related adverse events by treatment type, and patterns of first failure following radiotherapy to OPD lesions.

Outcomes were analyzed using Kaplan–Meier survival estimates and univariate and multivariate Cox proportional hazards models, with multivariate models incorporating covariates significant on univariate analysis. The Wilcoxon test was employed for comparisons between patient and treatment subgroups. Statistical significance was defined as *p* < 0.05, using log-rank tests to evaluate associations between clinical or treatment variables and LC, PFS, and OS. All analyses were conducted using R (v4.2.3), RStudio (v2022.12.0.353), and Microsoft Excel (v16.75).

## 3. Results

A total of 103 patients with 139 OPD lesions treated with radiotherapy were included. The cohort was predominantly female (n = 57, 55%), and the majority had a history of smoking (n = 84, 81%) (Table 1). Genomic profiling prior to 1st ICI was conducted in most patients (n = 92, 89%), revealing *KRAS* alterations as the most frequent molecular aberration (n = 37, 36%) (Supplementary Table S1). PD-L1 Tumor Proportion Score (TPS) was available for the majority of the cohort (n = 77, 75%) (Table 1). Most patients were initially diagnosed with advanced NSCLC with distant metastatic sites (n = 56, 54%), followed by patients with localized disease (n = 46, 45%) and unknown status (n = 1, 1%). Among localized NSCLC patients, 11 (24%) individuals remained with localized disease at the time of OPD. A total of 21 patients developed OPD of the primary lung lesion while on ICIs. The most frequent site of OPD was in the lungs (n = 33, 32%) or the brain (n = 26, 25%) (Table 2). OPD lesions were primarily either new lesions arising during ICI therapy (n = 45, 44%) or lesions that had previously demonstrated response or stability on ICI (n = 43, 42%) (Table 2). Most patients received treatment for a single OPD lesion (n = 79, 77%) (Table 2). The last ICI regimen administered prior to OPD-directed radiotherapy was most commonly anti-PD-1 monotherapy (n = 75, 73%) or in combination with chemotherapy, targeted agents, or anti-CTLA-4 agents (n = 15, 15%) (Supplementary Table S1). 

Median duration of ICI treatment before OPD diagnosis was 5.55 months (IQR = 2.07–10.56 months). The best overall response to ICI prior to OPD was most commonly partial response (PR) (n = 41, 40%) or stable disease (n = 32, 31%) (Table 2).

**Figure 1:**
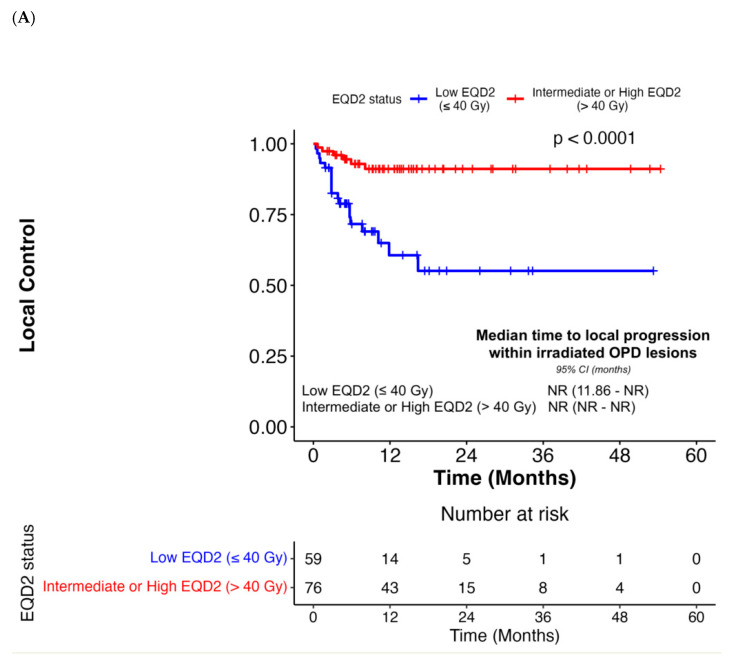

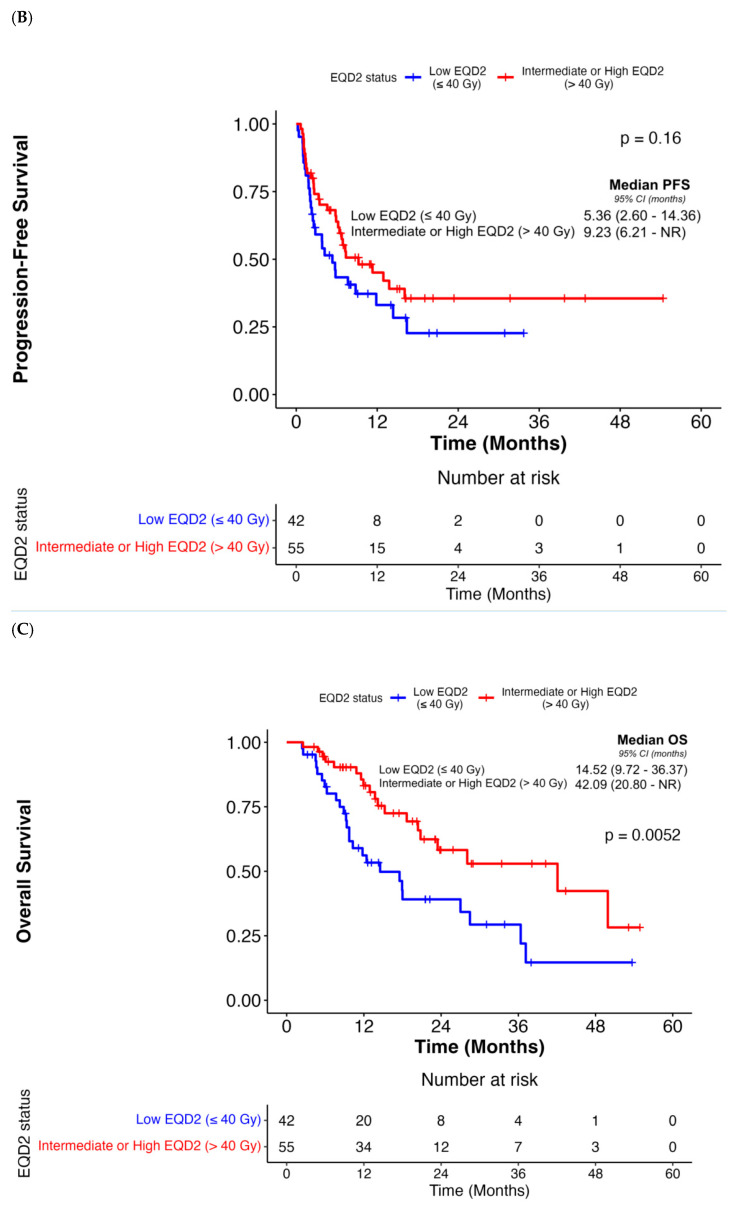
Associations between radiotherapy dose level using EQD2 with local control of irradiated oligoprogressive lesions (**A**), PFS (**B**), and OS (**C**). Abbreviations: Equivalent dose in 2 Gy fractions EQD2; OS, Overall Survival; PFS, Progression-Free Survival.

**Figure 2:**
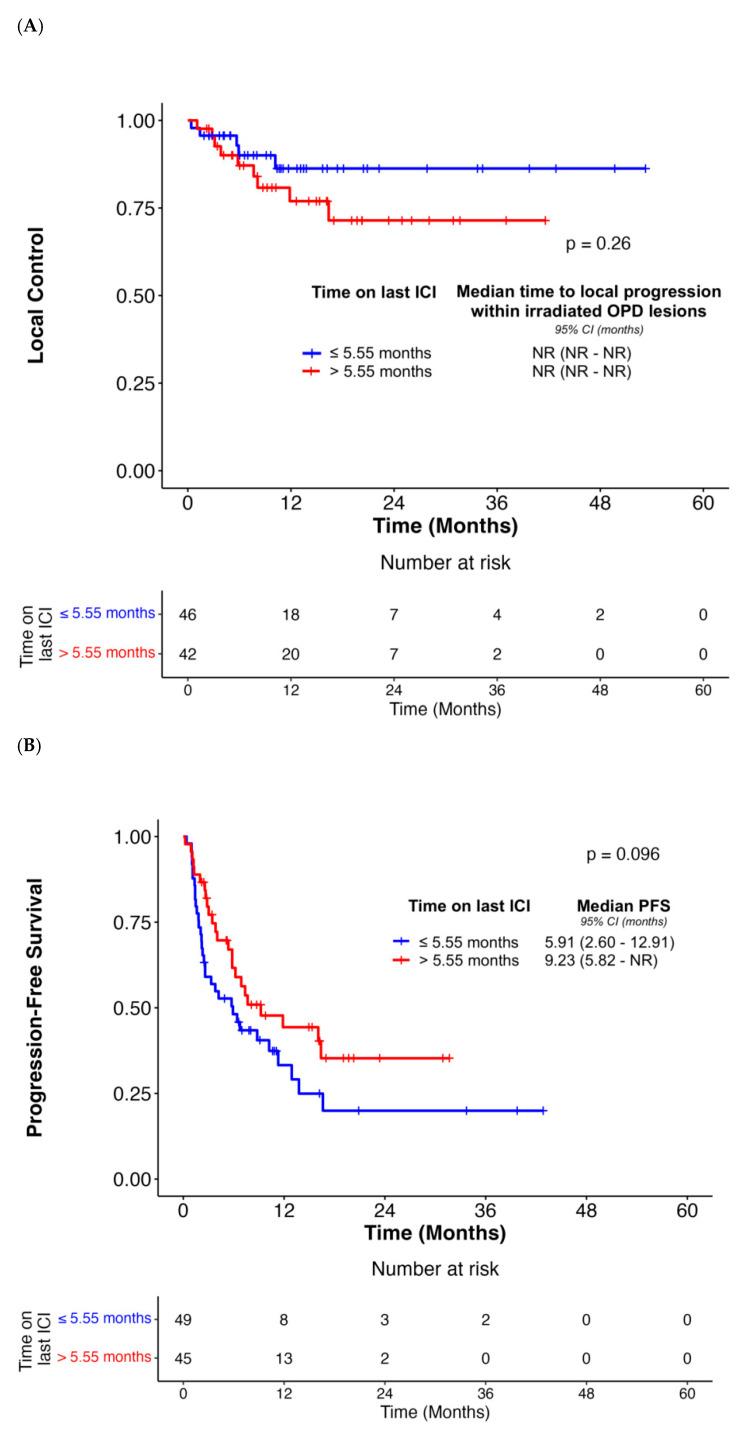

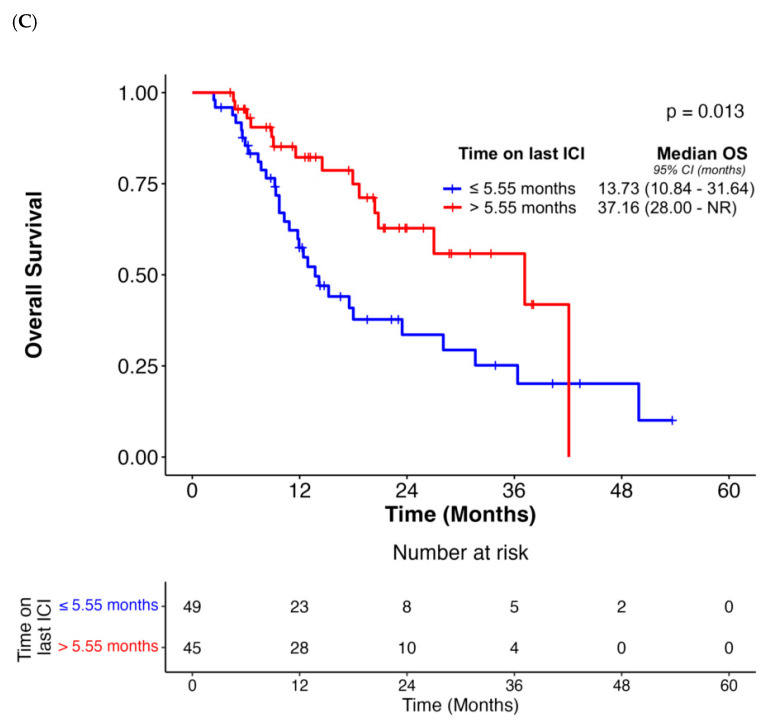
Associations between time on the most recent systemic ICI agent prior to OPD diagnosis and local control (**A**), PFS (**B**), and OS (**C**). The median value of 5.55 months was used to divide patients. Abbreviations: ICI, Immune Checkpoint Inhibitor; OS, Overall Survival; PFS, Progression-Free Survival.

Patients received various radiation modalities with varying fractionation schedules (Supplementary Table S2), with a similar proportion receiving stereotactic and non-stereotactic radiation at low or intermediate equivalent dose in 2 Gy fractions (EQD2) (Table 2). Radiotherapy achieved local control in most OPD lesions (n = 139), with a local response rate of 47% (n = 65) (Supplementary Table S3). Local failure was observed in only 10% of patients, with continued progression in the irradiated lesions (n = 14). The median duration of LC of radiated lesions was not reached (Supplementary Figure S1A); 23 (17%) irradiated lesions subsequently progressed/recurred after radiotherapy. Analysis of patient-specific factors did not demonstrate an association of LC with age at onset of OPD (*p* = 0.77), gender (*p* = 0.53), smoking status (*p* > 0.05), PD-L1 TPS (*p* = 0.15), and *KRAS* mutational status (*p* = 0.19) (Table 3). No significant associations were observed between LC-irradiated anatomic sites (*p* = 0.35) (Supplementary Figure S2A, Table 3, Supplementary Table S4). Subgroup analysis of brain OPD lesions revealed an excellent median LC with radiotherapy, which was not reached. Similarly, LC was not correlated with the number of irradiated OPD lesions (*p* = 0.84) or when comparing patients with cumulative OPD lesion volumes below the median (≤11.57 cm^3^) against those with a cumulative OPD lesion volume greater than 11.57 cm^3^ (*p* = 0.89) (Table 3). With respect to treatment-specific factors, no association was observed between LC and the radiation modality employed (*p* = 0.87). However, LC was significantly associated with delivery of intermediate or high EQD2 doses exceeding 40 Gy in adjusted models (*p* = 0.005) (Figure 1A, Table 4). Similarly, complete response (CR) or partial response of irradiated lesions was associated with the duration of LC, which remained significant in the multivariate analysis (*p* = 0.007) (Supplementary Figure S3A, Table 4). These responses were potentially driven by smaller OPD lesion volumes (*p* = 0.03), higher radiation dose per fraction (*p* < 0.001), and a hypofractionated radiotherapy regimen (*p* = 0.009) (Supplementary Figures S4–S6). There was no significant association between LC and either the best response of OPD lesions to prior ICI therapy (*p* > 0.05) or the duration of prior ICI therapy (*p* = 0.27) (Figure 2A, Table 4). Overall, treatment was well tolerated, with Grade 1–2 fatigue reported as the most common radiotherapy TRAE (n = 14, 14%). Additionally, 3 cases (3%) of non-severe radiation pneumonitis were observed. Regarding severe toxicities, pneumonitis was the most commonly occurring TRAE attributed to ICIs (n = 5, 5%) (Supplementary Table S5). After completion of radiotherapy, 67 (65%) patients resumed systemic treatment with the same ICI regimen. Local management following the onset of 2nd OPD involved radiotherapy (n = 23) and surgery (n = 1). In this radiotherapy cohort, 15 patients had continued ICIs after their 1st OPD before eventually developing a 2nd OPD, which warranted further radiation.

The median PFS was 6.90 months (95% CI, 5.75–12.91) for all patients (Supplementary Figure S1B). This included a subset of NSCLC patients (n = 9) with prolonged PFS (range = 13.77–39.72 months) while off all systemic therapy following radiation. These patients exhibiting extended immune-mediated tumor control were treated with stereotactic (n = 6) and conventional (n = 3) radiotherapy, with local response noted in 4 patients (1 complete response, 3 partial responders). Analysis of patterns of first progression, whether local or distant to the irradiated site, revealed that among the 64 patients who subsequently progressed (62% of the cohort), progression most frequently occurred in pre-existing lesions that had previously been responsive or stable on the last ICI therapy (n = 17, 27%) or developed new lesions (n = 16, 25%) (Supplementary Table S6). There were no statistically significant associations between PFS and age at OPD diagnosis (*p* = 0.17), gender (*p* = 0.11), smoking status (*p* > 0.05), and PD-L1 TPS (*p* = 0.94) (Table 3). *KRAS* mutational status was also not associated with PFS in the adjusted models (*p* = 0.11) (Table 3). In contrast, multivariate analysis demonstrated that irradiation of visceral OPD sites was associated with improved PFS (*p* = 0.01) (Supplementary Figure S2B, Table 3). Subgroup analysis of brain OPD lesions demonstrated a median PFS of 6.41 months (95% CI, 3.35—not reached). There were no associations identified between PFS and the number of OPD lesions (*p* = 0.54) or cumulative OPD lesion volume (*p* = 0.89) (Table 3). We also did not observe an association of PFS with radiotherapy-specific factors, including radiation modality (*p* = 0.47) or EQD2 status (*p* = 0.16) (Figure 1B, Table 4). On univariate analysis, local response in irradiated OPD lesions was associated with PFS; however, this association was not significant in multivariate models (*p* = 0.15) (Supplementary Figure S3B, Table 4). In addition, PFS was not linked to the best response of OPD lesions to prior ICI therapy (*p* > 0.05) or to the duration of the last ICI before onset of OPD (*p* = 0.10) (Figure 2B, Table 4).

In the complete cohort, median OS after OPD treatment was 23.46 months (95% CI, 17.54–37.16) (Supplementary Figure S1C). There were no associations between OS and patient-specific factors comprising age at onset of OPD, gender, smoking status, PD-L1 TPS, *KRAS* mutational status, anatomic site (Supplementary Figure S2C), number of OPD lesions, and cumulative OPD lesion volumes (*p* > 0.05 for all) (Table 3). Patients harboring brain OPD lesions demonstrated a median OS of 28.06 months (95% CI, 11.76—not reached). In terms of treatment-specific factors, although radiation modality was not associated with OS (*p* = 0.06), NSCLC patients receiving intermediate or high EQD2 of >40 Gy were associated with an improvement in OS outcomes in the adjusted models (*p* = 0.01) (Figure 1C, Table 4). We also observed that OS was associated with local response in irradiated OPD lesions in the multivariate analysis (*p* = 0.006) (Supplementary Figure S3C, Table 4). Conversely, we did not note an association between OS and the best response of OPD lesions to prior ICI therapy (*p* > 0.05) (Table 4). Finally, multivariate analysis demonstrated that NSCLC patients receiving ICIs for a duration of >5.55 months prior to onset of OPD were associated with a favorable OS outcome (Figure 2C, Table 4).

## 4. Discussion

The increasing number of cancer patients receiving ICIs has led to more patients with either primary or acquired ICI resistance. Management of limited progression after ICI therapy remains unclear, though increasing evidence suggests some patients benefit from continued ICIs after local ablation or surgical removal of resistant disease [17,18]. This approach may provide a substantial clinical benefit, as subsequent lines of systemic therapy are often associated with shorter durations of response and increased toxicity relative to ICIs. However, the identification of patients with true OPD who are suitable for local therapy remains complex, and there is a paucity of data regarding which individuals experience the most favorable outcomes from local treatment of OPD. To address this question in the context of NSCLC, we reviewed outcomes for such patients developing OPD during ICI therapy and who received radiotherapy without starting a new systemic treatment regimen. To our knowledge, this review represents the largest analysis to date of NSCLC patients treated with radiotherapy after OPD on ICIs.

Our results support the application of this therapeutic strategy in well-selected patients, demonstrating favorable LC and encouraging durations of PFS and OS. These observations align with emerging evidence from the Phase II CURB OPD trial, which showed that NSCLC patients treated with combined modalities achieved a significant PFS advantage compared with individuals receiving standard systemic therapy alone, including immunotherapy [18]. Of note, 39% of patients in the stereotactic body radiotherapy (SBRT) cohort had received radiotherapy to more than 5 oligoprogressive lesions, with no subsequent progression of irradiated OPD lesions in the SBRT arm. Most patients in the CURB trial received an EQD2 ≥ 50 Gy, which is concordant with our results, where patients receiving intermediate- to high-dose radiotherapy (i.e., EQD2 > 40 Gy) were associated with improved LC and OS. Prior studies suggest that irradiation of oligometastasis followed by ICI can result in favorable outcomes [19]. Here, we describe a complementary treatment strategy for a cohort of patients with OPD who had already initiated prior ICI therapy. This approach has been examined in NSCLC populations receiving local ablative therapy in combination with various systemic treatments, with retrospective analyses demonstrating favorable outcomes despite limited sample sizes [20,21]. Collectively, these findings have contributed to the incorporation of local ablative therapies into NSCLC treatment guidelines [22].

As expected, LC was associated with local response. We also found local response to be associated with OS irrespective of the number of OPD lesions, highlighting the possible contribution of effective local therapy to disease-specific outcomes. Treatment response was potentially driven by small OPD lesion volumes and delivery of higher doses of hypofractionated radiotherapy. Interestingly, we observed that the benefit of radiotherapy in OPD is not restricted to high-PD-L1 expressors. Hence, management of small oligoprogressive lesions with hypofractionated, ablative treatment such as SBRT could be an ideal treatment option, where feasible, likely irrespective of PD-L1 score. The shorter duration of treatment would add to patient convenience, increase healthcare savings, and potentially improve survival outcomes while minimizing delays in ICI administration [23].

Patterns of out-of-field failure following radiation were heterogeneous and mostly included progression of pre-existing lesions or development of new lesions. With respect to the CURB trial, the incidence of progression (after radiation of OPD lesions) of previously responsive or stable lesions on systemic therapy was similar in both studies. However, we observed a relatively lower proportion of patients developing new lesions in our study.

Our additional findings that improved PFS was associated with visceral OPD sites, and that better OS correlated with longer duration of prior ICI could potentially help guide patient selection. Patients whose tumors have non-visceral OPD lesions or who progress more rapidly on prior ICIs may be more likely to harbor micrometastatic disease not detectable on imaging or may demonstrate systemic immune escape rather than isolated areas of ICI resistance. Circulating tumor DNA (ctDNA) can be useful in this instance, as the CURB trial demonstrated that ctDNA fraction and mutant allele frequencies from patients’ blood samples correlated with NSCLC disease burden and SBRT treatment.

Additionally, the association between longer duration of prior ICI and improved OS in OPD patients suggests the presence of a clinically distinct subgroup characterized by partial but durable systemic disease control. This improvement in patient survival may reflect a more sustained and effective immune-tumor interaction. Extended delivery of ICIs before OPD onset may enable a gradual and durable activation of T cell-mediated immunity, promoting enhanced tumor surveillance prior to immune dysregulation [24,25]. Immune system “conditioning” over time through ICI treatment may further aid maturation and expansion of tumor-reactive T cells, contributing to durable clinical benefit [26,27]. In these patients, progression appears to be limited following an initial period of ICI treatment benefit, rather than as widespread systemic failure. Within this context, radiotherapy may be considered as a means of addressing resistant sites of disease while overall systemic control is maintained, rather than as a strategy expected to overcome generalized resistance to ICIs. Importantly, these findings should not be interpreted as evidence of uniform efficacy of ICIs across patients with advanced NSCLC. A notable proportion of our patients experienced primary resistance or early acquired resistance to ICIs, resulting in limited duration of benefit and early disease progression despite treatment. In this context, disease progression is more often diffuse rather than oligoprogressive, reflecting generalized treatment failure, and such patients are therefore less likely to derive meaningful clinical benefit from local interventions such as radiotherapy, thereby aiding physicians to consider other treatment options and to avoid radiation-specific toxicities.

Moreover, the absence of an association between PD-L1 TPS and survival in this cohort, despite a correlation with prior ICI duration, underscores the limitations of PD-L1 as a standalone predictive biomarker. Duration of ICI may instead serve as a pragmatic, post-hoc indicator of clinical benefit that integrates multiple factors—including tumor biology, host immune response, and treatment tolerance—that are not captured by PD-L1 expression alone. This finding is consistent with prior observations that durable responses to ICIs can occur across PD-L1 expression levels [28,29]. Importantly, this observation does not diminish the role of PD-L1 in treatment selection but rather highlights the need for additional or composite biomarkers to better predict long-term benefit from ICIs.

Overall, the treatment was well tolerated, with a few instances of non-severe radiation pneumonitis. Future investigations would benefit from the exploration of radiation doses to the lung and their impact on radiation pneumonitis incidence in the context of OPD management.

Our study is limited by heterogeneity in prior treatments, as well as in the number, volume, and site of OPD lesions, in addition to variability in radiotherapy doses and schedules. The limited sample sizes within subgroups may also have reduced statistical power for subgroup analyses of survival outcomes. Our study did not account for baseline performance status prior to radiotherapy, underlying tumor biology, or treatment sensitivity, which could have affected study outcomes. Another limitation is the lack of biological correlates, as emerging evidence suggests the increasing role of utilizing ctDNA to identify NSCLC candidates likely to develop OPD while on systemic agents [30].

## 5. Conclusions

Our data contributes to aiding multidisciplinary clinical decisions advocating for radiotherapy use among patients with OPD on ICIs in select cases. Clinical factors, including the presence of visceral OPD lesions and the duration of prior ICI therapy, may aid in refining patient selection. Treatment of smaller OPD lesions using intermediate- to high-dose, hypofractionated radiotherapy can promote improved local response while maintaining a low toxicity profile. Attaining local control appears to be associated with an improvement in PFS and OS outcomes and may postpone transition to more toxic systemic therapies. These findings should be interpreted as hypothesis-generating and require validation in prospectively designed clinical trials.

## Figures and Tables

**Table 1:** Baseline characteristics of evaluated patients.

	Total Number of Patients (N = 103)	
	N	%
**Age at OPD diagnosis (years)**		
Median	68	
Range	44–90	
Interquartile range	62–74	
**Gender**		
Male	46	45%
Female	57	55%
**Smoking Status**		
Never Smoker	15	15%
Current Smoker	20	19%
Former Smoker	64	62%
Unknown	4	4%
PD-L1 TPS Status *		
<1%	18	18%
1–49%	18	18%
≥50%	41	40%

Abbreviations: OPD, oligoprogressive disease; PD-L1, programmed death-ligand 1; TPS, tumor proportion score. * Available results (N = 77, 75%), unknown results (N = 26, 25%).

**Table 2:** Treatment details of OPD lesions among evaluated patients.

	Total Number of Patients (N = 103 *)	
	N	%
**Anatomic location of radiated OPD lesions**		
Lung	33	32%
Brain	26	25%
Lymph node	10	10%
Spine	10	10%
Bone	8	8%
Other sites **	16	16%
**Duration of treatment with last ICI before OPD diagnosis (months)**		
Median	5.55	
Interquartile range	2.07–10.56	
**Best overall response to last ICI**		
Complete Response	4	4%
Partial Response	41	40%
Mixed response	2	2%
Stable Disease	32	31%
Progressive Disease	15	15%
Unevaluable	9	9%
**Response of OPD lesions to prior ICI**		
Patients with new lesions	45	44%
Patients with lesions previously responsive or stable on ICI	43	42%
Patients with lesions that never responded to ICI	14	14%
Patients with new lesions and lesions that never responded to ICI	1	1%
**Number of OPD lesions**		
Patients with 1 OPD lesion	79	77%
Patients with 2 OPD lesions	18	17%
Patients with >2 OPD lesions	6	6%
**Radiation modality used to treat OPD lesions**		
SABR/SRS/SRT	49	48%
Non-stereotactic RT	50	49%
Other radiation modality ^†^	4	4%
**Radiation total dose by EQD2 levels**		
Low EQD2 (≤40 Gy)	42	41%
Intermediate EQD2 (>40 Gy and <80 Gy)	40	39%
High EQD2 (≥80 Gy)	15	15%
Low and intermediate EQD2	6	6%
**Re-irradiation of OPD lesions**		
Yes	6	6%
No	97	94%

Abbreviations: EQD2, equivalent dose; Gy, Gray; ICI, immune checkpoint inhibitor; OPD, oligoprogressive disease; RT, radiotherapy; SABR, stereotactic Ablative Radiotherapy; SRS, stereotactic radiosurgery; SRT; stereotactic radiation therapy. * Note: All data is presented on a per-patient basis (N = 103) for a total of 139 lesions. ** Other sites: Adrenal gland (N = 6), lung and lymph node (N = 2), lung and bone (N = 1), lung and spine (N = 1), liver (N = 1), pancreas (N = 1), adrenal gland and lymph node (N = 1), muscle (N = 1), muscle and lymph node (N = 1), and upper arm and supraclavicular region (N = 1). ^†^ Other radiation modalities: whole brain radiotherapy (N = 2), SBRT + Adaptive IMRT (N = 1), and SBRT + Conformal (N = 1).

**Table 3:** Associations of patient-specific factors with LC, PFS, and OS outcomes.

	LC				PFS				OS			
	Univariate HR (95% CI)	*p*-Value	Adjusted HR (95% CI) *	*p*-Value	Univariate HR (95% CI)	*p*-Value	Adjusted HR (95% CI) *	*p*-Value	Univariate HR (95% CI)	*p*-Value	Adjusted HR (95% CI) *	*p*-Value
**Age at OPD diagnosis**	1.00 (0.94–1.04)	0.77	N/A	N/A	0.98 (0.96–1.01)	0.17	N/A	N/A	1.01 (0.98–1.04)	0.50	N/A	N/A
**Gender (Male vs. Female)**	1.36 (0.52–3.53)	0.53	N/A	N/A	1.50 (0.91–2.47)	0.11	N/A	N/A	1.85 (1.04–3.28)	0.04	N/A	N/A
**Smoking status**												
Current Smoker	-	-	-	-	-	-	-	-	-	-	-	-
Former Smoker	0.79 (0.25–2.51)	0.68	N/A	N/A	1.13 (0.60–2.16)	0.70	N/A	N/A	0.90 (0.45–1.80)	0.76	N/A	N/A
Never Smoker	1.05 (0.24– 4.72)	0.95	N/A	N/A	1.39 (0.60–3.23)	0.44	N/A	N/A	1.14 (0.45–2.87)	0.78	N/A	N/A
**PD-L1 TPS status** **(≥50** **% vs. <50%)**	2.67 (0.71–10.09)	0.15	N/A	N/A	0.98 (0.53–1.79)	0.94	N/A	N/A	1.19 (0.57–2.50)	0.64	N/A	N/A
***KRAS*** ** (Mutant vs. wild type)**	0.42 (0.12–1.53)	0.19	N/A	N/A	0.52 (0.30–0.92)	0.02	0.62 (0.35–1.11)	0.11	0.73 (0.39–1.37)	0.33	N/A	N/A
**Anatomic location of OPD lesions (Visceral vs. non-visceral)**	0.64 (0.24–1.66)	0.35	N/A	N/A	0.56 (0.32–0.99)	0.046	0.45 (0.24–0.83)	**0.01**	0.73 (0.39–1.37)	0.32	N/A	N/A
**Number of OPD lesions**	1.06 (0.60–1.86)	0.84	N/A	N/A	1.11 (0.82–1.47)	0.54	N/A	N/A	0.95 (0.68–1.33)	0.77	N/A	N/A
**Median cumulative OPD lesion volume (≤11.57 cm^3^ vs. >11.57 cm^3^)**	1.05 (0.55–1.98)	0.89	N/A	N/A	1.09 (0.33–3.58)	0.89	N/A	N/A	0.52 (0.25–1.08)	0.08	N/A	N/A

Abbreviations: HR, Hazard Ratio; LC, Local Control; N/A, Not Applicable; OS, Overall Survival; OPD, Oligoprogressive Disease; PD-L1, Programmed Death-Ligand 1; PFS, Progression-Free Survival; TPS, Tumor Proportion Score. * Adjusted analysis by age, gender, smoking status, tumor subtype, and number of OPD lesions. Red font: Most relevant statistically significant result in the adjusted analysis.

**Table 4:** Associations of treatment-specific factors with LC, PFS, and OS outcomes.

	LC				PFS				OS			
	Univariate HR (95% CI)	*p*-Value	Adjusted HR (95% CI) *	*p*-Value	Univariate HR (95% CI)	*p*-Value	Adjusted HR (95% CI) *	*p*-Value	Univariate HR (95% CI)	*p*-Value	Adjusted HR (95% CI) *	*p*-Value
**Radiation modality (SABR/SRS vs. Conventional RT)**	0.94 (0.42–2.07)	0.87	N/A	N/A	0.83 (0.50–1.37)	0.47	N/A	N/A	0.57 (0.32–1.03)	0.06	N/A	N/A
**EQD2 status (α/β = 10)**												
Low EQD2 (≤40 Gy)	-	-	-	-		-	-	-	-	-	-	-
Intermediate or High EQD2 (>40 Gy)	0.19 (0.07–0.47)	< 0.001	0.14 (0.04–0.56)	**0.005**	0.69 (0.41–1.16)	0.16	N/A	N/A	0.44 (0.24–0.79)	0.007	0.35 (0.16–0.80)	**0.01**
**Local treatment response to RT in OPD lesions (Responders vs. Non-Responders)**	0.21 (0.08–0.53)	< 0.001	0.07 (0.01–0.48)	**0.007**	0.55 (0.32–0.96)	0.04	0.65 (0.37–1.17)	0.15	0.47 (0.24–0.91)	0.03	0.38 (0.19–0.76)	**0.006**
**Response of OPD lesions to prior ICI**												
Previously responsive or stable on ICI	-	-	-	-		-	-	-	-	-	-	-
Never responded to ICI	1.01 (0.28–3.62)	0.99	N/A	N/A	1.10 (0.52–2.32)	0.81	N/A	N/A	1.54 (0.72–3.29)	0.27	N/A	N/A
New lesions	0.76 (0.33–1.76)	0.53	N/A	N/A	0.84 (0.48–1.45)	0.52	N/A	N/A	0.83 (0.44–1.57)	0.58	N/A	N/A
**Median duration of last ICI before OPD diagnosis (>5.55 months vs. ≤5.55 months)**	1.86 (0.62–5.56)	0.27	N/A	N/A	0.64 (0.38–1.09)	0.10	N/A	N/A	0.46 (0.25–0.87)	0.02	0.47 (0.24–0.93)	**0.03**

Abbreviations: EQD2, Equivalent Dose in 2 Gy fractions; HR, Hazard Ratio; ICI, Immune Checkpoint Inhibitor; LC, Local Control; N/A, Not Applicable; OS, Overall Survival; OPD, Oligoprogressive Disease; PFS, Progression-Free Survival; RT, Radiotherapy; SABR, Stereotactic ablative radiotherapy; SRS, Stereotactic Radiosurgery. * Adjusted analysis by age, gender, smoking status, and number of OPD lesions. Red font: Most relevant statistically significant result in the adjusted analysis.

## Data Availability

The original contributions presented in this study are included in the article/Supplementary Materials; further inquiries can be directed to the corresponding authors.

## Supplementary Materials

The following supporting information can be downloaded at: https://www.mdpi.com/article/10.3390/cancers18010071/s1, Figure S1: An overview of survival outcomes in the complete dataset comprising of further progression within radiated OPD lesion(s) (A), PFS based on the first progression event at any site following radiation (B) and OS (C); Figure S2: Associations between anatomic sites of OPD with local control of irradiated oligoprogressive lesions (A), PFS (B) and OS (C); Figure S3: Associations between local response of irradiated OPD lesions and local control (A), PFS (B) and OS (C). Patients achieving complete or partial response were classified as responders whereas patients attaining stable disease or progressive disease as their local response were categorized as non-responders; Figure S4: Boxplot of patients’ OPD lesion volume with respect to their best local response to radiotherapy; Figure S5: Boxplot of radiation dose per fraction with respect to the best local response of OPD lesions to radiotherapy; Figure S6: Boxplot of number of radiotherapy fractions with respect to the best local response of OPD lesions to radiotherapy; Table S1: Mutation profile, previous lines of treatment and types of ICI prior to radiation for oligoprogressive disease among evaluated patients; Table S2: Fractionation schedule for each radiated oligoprogressive lesion among patients receiving stereotactic, non-stereotactic and other radiation modalities; Table S3: Best local response of each radiated oligoprogressive lesion; Table S4: Best local response of each radiated oligoprogressive lesion among patients receiving stereotactic, non-stereotactic and other radiation modalities; Table S5: Severe treatment related adverse events attributed to immune checkpoint inhibitors; Table S6: Patterns of first failure following radiation to oligoprogressive lesions among treated patients (N = 64).
