# Supplementary material for: Outcomes Following Radiotherapy for Oligoprogressive NSCLC on Immune Checkpoint Inhibitors: A Real-World, Multinational Experience
Source: Cancers (Basel). 2025 Dec 25;18(1):71. doi: 10.3390/cancers18010071 (PMC12784812; doi:10.3390/cancers18010071)
Supplement: Supplementary file 1 [file cancers-18-00071-s001.zip › cancers-4042206-supplementary/Supplementary Figures.pdf]

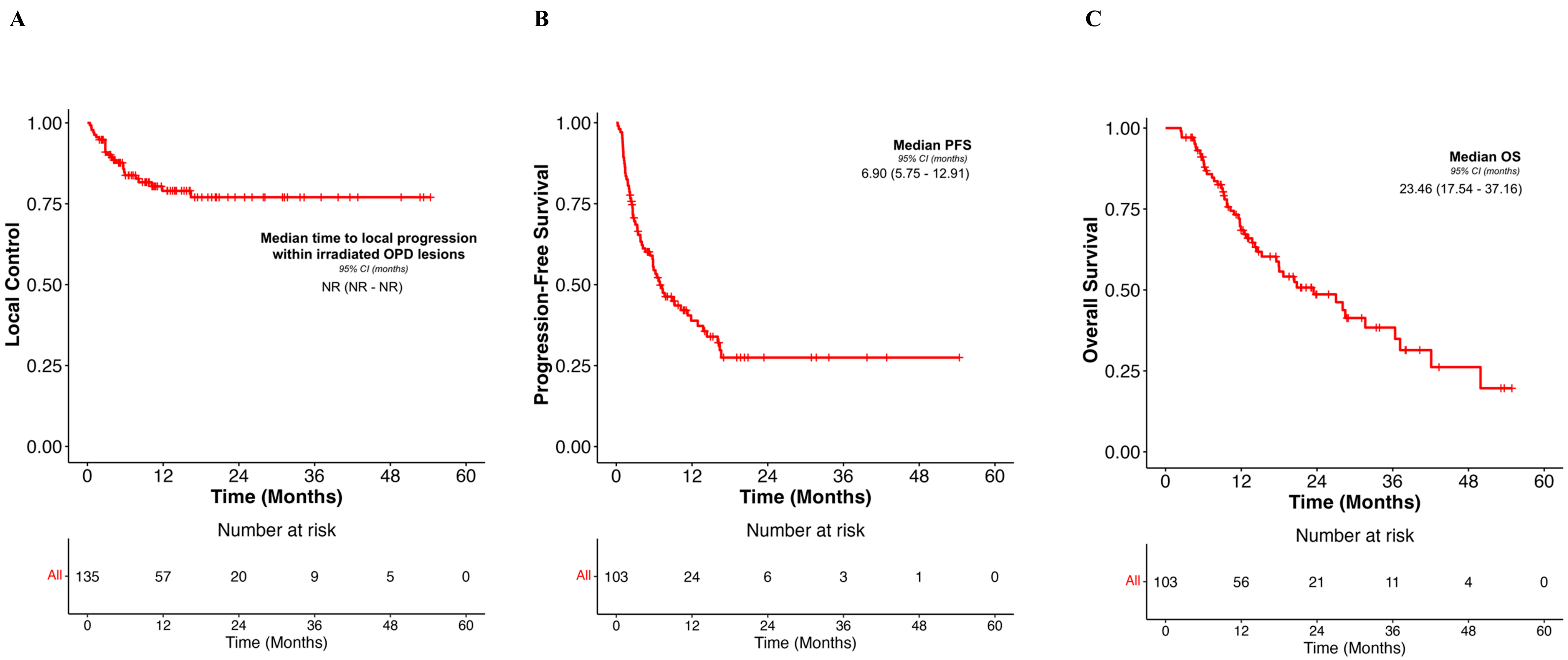

Supplementary Figure S1: An overview of survival outcomes in the complete dataset comprising of further progression within radiated OPD lesion(s) (A), PFS based on the first progression event at any site following radiation (B) and OS (C).

Abbreviations: OPD, Oligoprogressive Disease; OS, Overall Survival; PFS, Progression Free Survival.

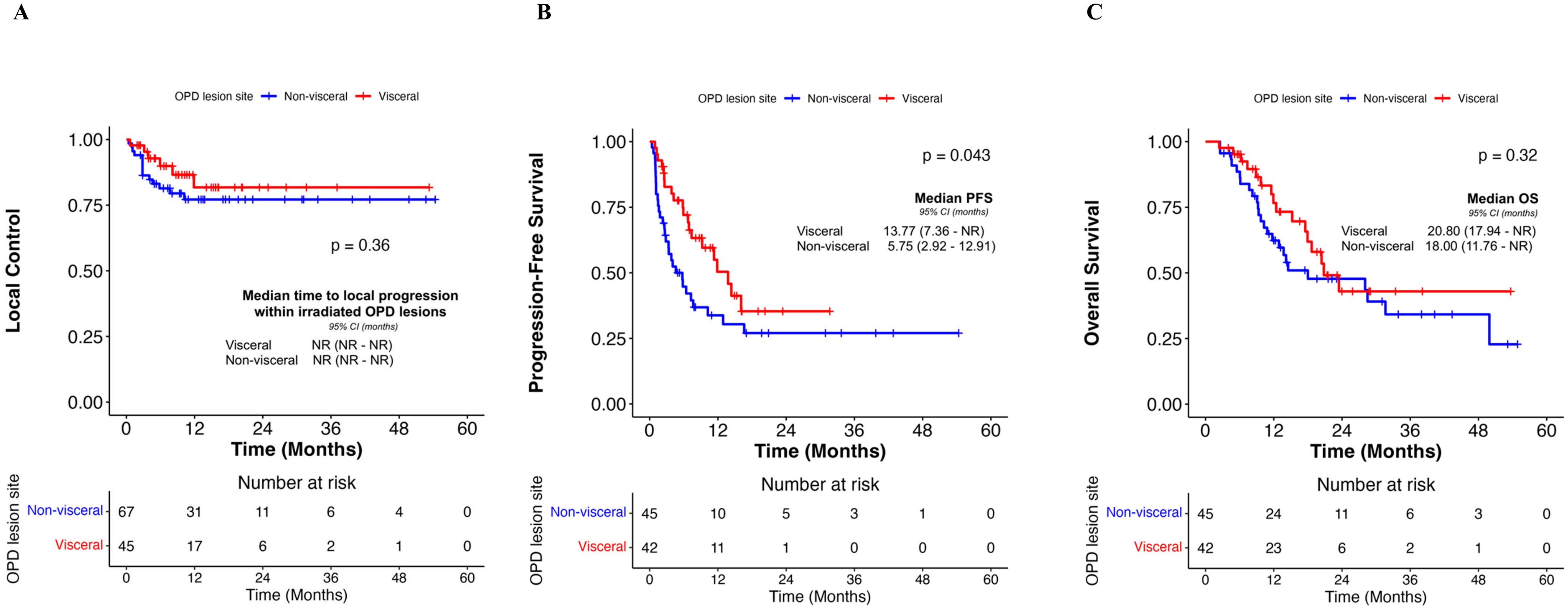

Supplementary Figure S2: Associations between anatomic sites of OPD with local control of irradiated oligoprogressive lesions (A), PFS (B) and OS (C).

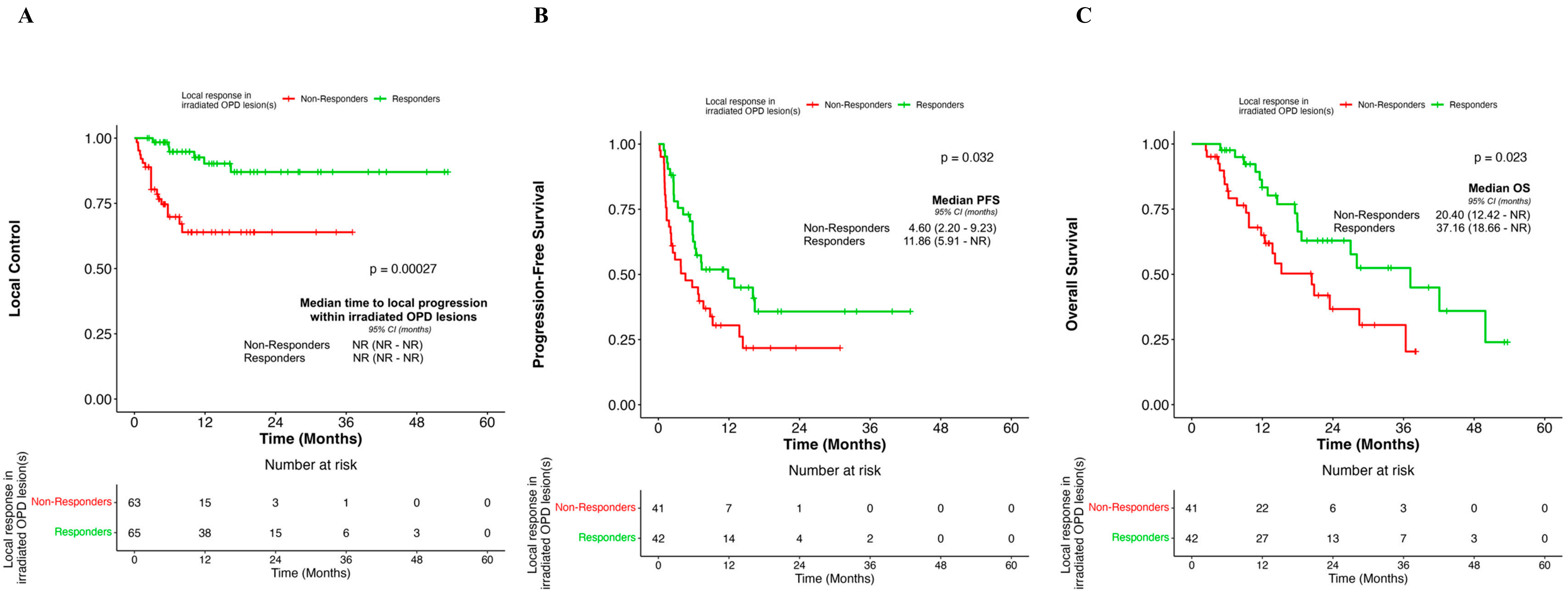

Supplementary Figure S3: Associations between local response of irradiated OPD lesions and local control (A), PFS (B) and OS (C). Patients achieving complete or partial response were classified as responders whereas patients attaining stable disease or progressive disease as their local response were categorized as non-responders.

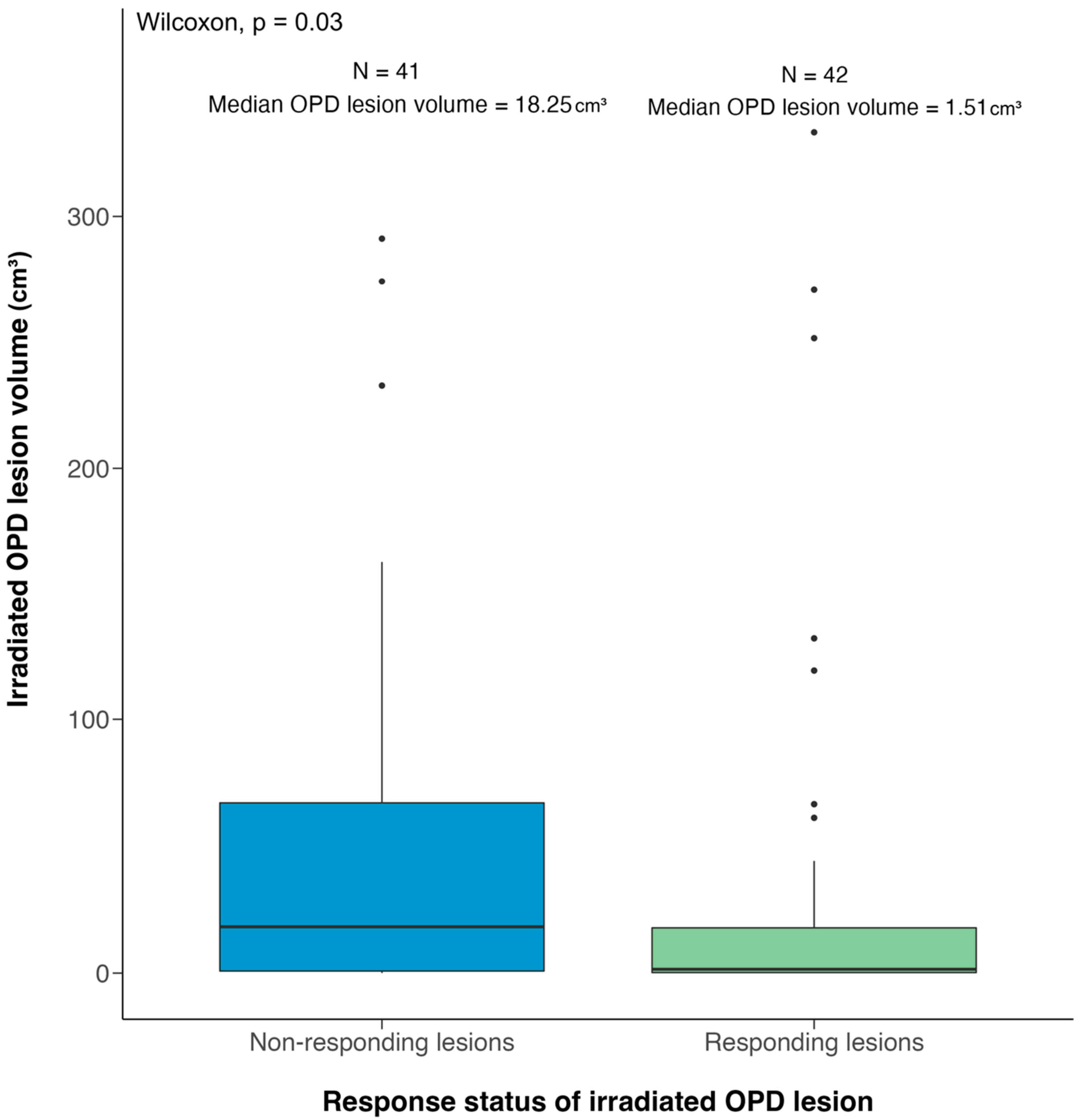

Supplementary Figure S4: Boxplot of patients' OPD lesion volume with respect to their best local response to radiotherapy.

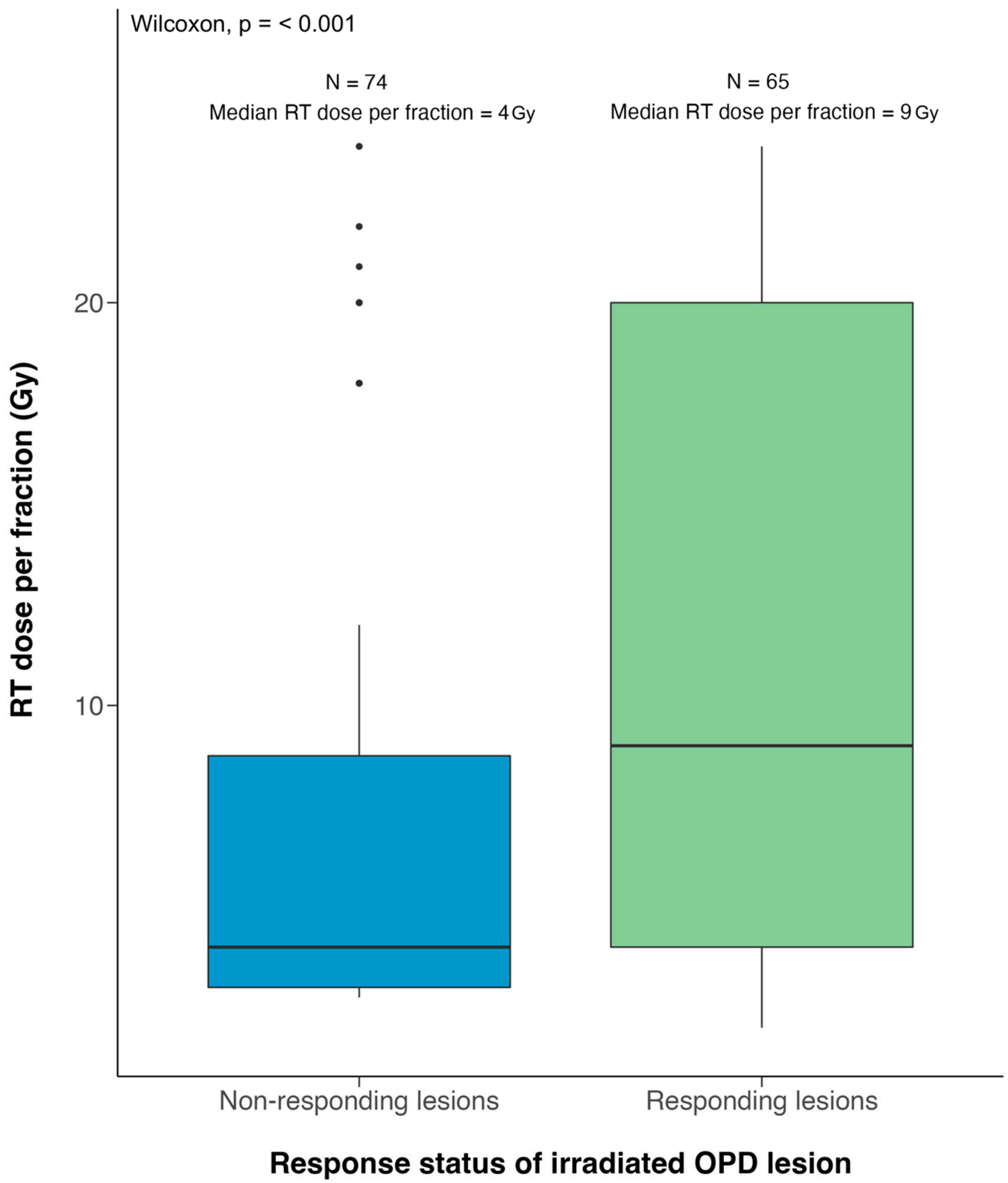

Supplementary Figure S5: Boxplot of radiation dose per fraction with respect to the best local response of OPD lesions to radiotherapy.

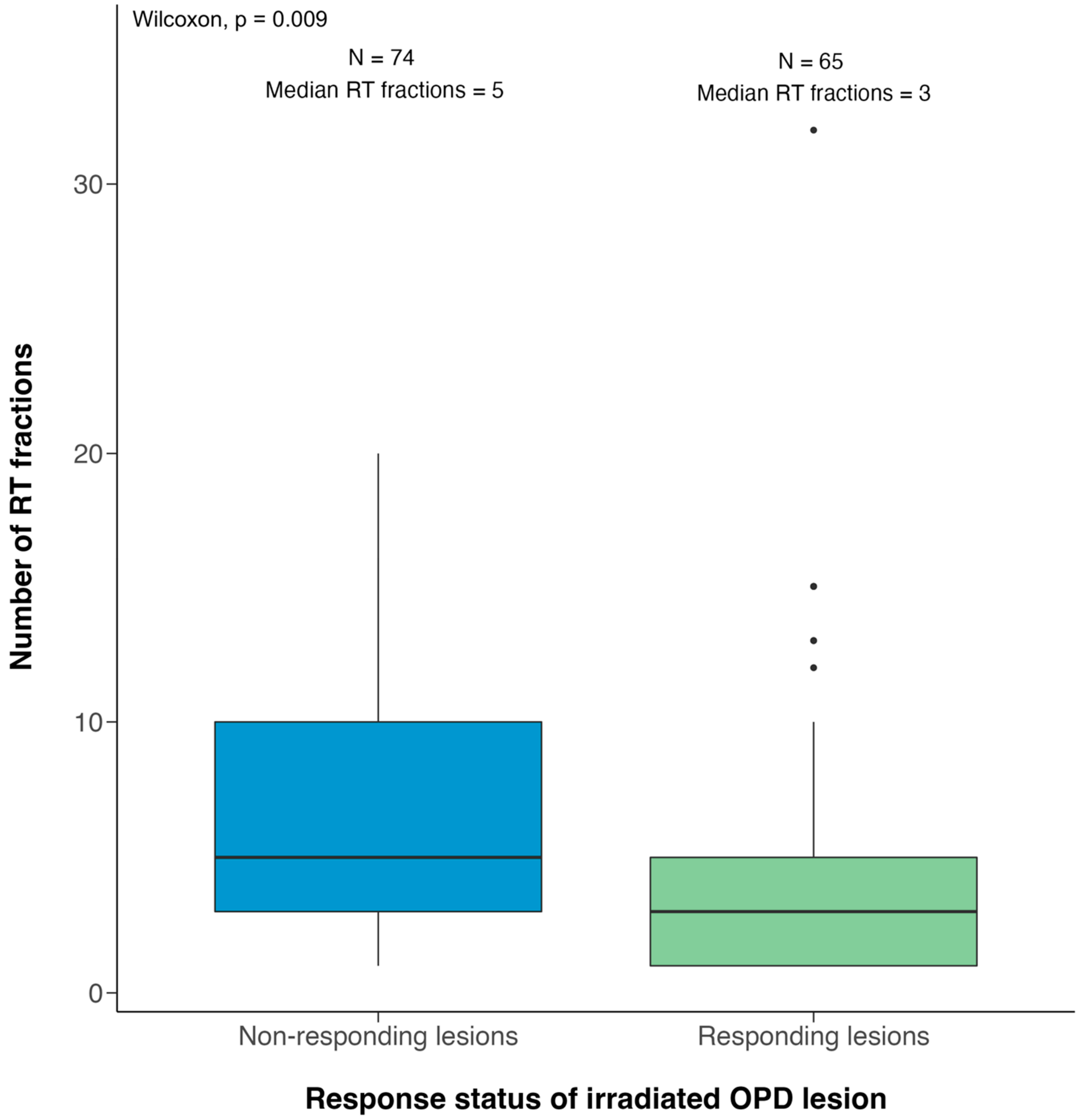

Supplementary Figure S6: Boxplot of number of radiotherapy fractions with respect to the best local response of OPD lesions to radiotherapy.
