# Supplementary material for: Outcomes Following Radiotherapy for Oligoprogressive NSCLC on Immune Checkpoint Inhibitors: A Real-World, Multinational Experience
Source: Cancers (Basel). 2025 Dec 25;18(1):71. doi: 10.3390/cancers18010071 (PMC12784812; doi:10.3390/cancers18010071)
Supplement: Supplementary file 1 [file cancers-18-00071-s001.zip › cancers-4042206-supplementary/Supplementary Tables.pdf]

Supplementary Tables

Supplementary Table S1: Mutation profile, previous lines of treatment and types of ICI prior to radiation for oligoprogressive disease among evaluated patients.

	**Total number of patients (N = 103)**	
	**N**	**%**
**Mutation status**		
EGFR +	7	7%
EGFR -	73	71%
Unknown EGFR status	23	22%
ALK +	1	1%
ALK -	80	78%
Unknown ALK status	22	21%
KRAS +	37	36%
KRAS -	50	49%
Unknown KRAS status	16	15%
BRAF +	3	3%
BRAF -	66	64%
Unknown BRAF status	34	33%
TP53 +	18	17%
TP53 -	42	41%
Unknown TP53 status	43	42%
**Prior treatment**		
Any cancer related surgery	34	33%
Radiation therapy	42	41%
Chemotherapy	69	67%
Targeted therapy	5	5%
**ICI immediately preceding RT for OPD**		
Anti-PD-1 alone	75	73%
Anti-PD-1 combination*	15	15%
Anti-PD-L1 alone	11	11%
Anti-PD-L1 combination[†]	2	2%

Abbreviations: ALK, anaplastic lymphoma kinase; BRAF, v-raf murine sarcoma viral oncogene homolog B1; CTLA-4, cytotoxic T lymphocyte antigen-4; EGFR, epidermal growth factor receptor; ICI, immune checkpoint inhibitor; KRAS, kirsten rat sarcoma viral oncogene; OPD, oligoprogressive disease; PD-1, programmed cell death-1; PD-L1, programmed death-ligand 1; TP53, tumor protein p53.

*Anti-PD-1 combination: anti-PD-1 and chemotherapy (N = 12), anti-PD-1 and targeted therapy (N = 2), anti-PD-1 and anti-CTLA-4 therapy (N = 1).

† Anti-PD-L1 combination: anti-PDL-1 and anti-CTLA-4 therapy with LAG-3 inhibition (N = 1), anti-PD-L1 and targeted therapy (N = 1).

Supplementary Table S2: Fractionation schedule for each radiated oligoprogressive lesion among patients receiving stereotactic, non-stereotactic and other radiation modalities.

	**Total number oligoprogressive lesions (N = 139)**	
	**N**	**%**
**SBRT / hypofractionated RT (N = 28)**		
25 Gy (5Gy x 5 fractions)	1	4
30 Gy (6Gy x 5 fractions)	3	11
30 Gy (10Gy x 3 fractions)	2	7
32 Gy (8Gy x 4 fractions)	2	7
35 Gy (7Gy x 5 fractions)	1	4
36 Gy (12Gy x 3 fractions)	2	7
45 Gy (9Gy x 5 fractions)	2	7
48 Gy (16Gy x 3 fractions)	1	4
50 Gy (10Gy x 5 fractions)	3	11
54 Gy (18Gy x 3 fractions)	4	14
55 Gy (11Gy x 5 fractions)	1	4
60 Gy (7.5Gy x 8 fractions)	1	4
60 Gy (12Gy x 5 fractions)	5	18
**SRS/SRT, including hypofractionated regimens (N = 43)**		
18 Gy (18Gy x 1 fractions)	2	5
20 Gy (20Gy x 1 fractions)	18	42
21 Gy (7Gy x 3 fractions)	7	16
21 Gy (21Gy x 1 fractions)	1	2
22 Gy (22Gy x 1 fractions)	1	2
24 Gy (8Gy x 3 fractions)	3	7
24 Gy (24Gy x 1 fractions)	9	21

25 Gy (5Gy x 5 fractions)	1	2
27 Gy (9Gy x 3 fractions)	1	2
**Non-stereotactic RT (N = 62)**		
8 Gy (8Gy x 1 fractions)	7	11
20 Gy (4Gy x 5 fractions)	10	16
20 Gy (5Gy x 5 fractions)	2	3
24 Gy (4Gy x 6 fractions)	2	3
25 Gy (5Gy x 5 fractions)	3	5
30 Gy (3Gy x 10 fractions)	15	24
36 Gy (3Gy x 12 fractions)	2	3
39 Gy (3Gy x 13 fractions)	11	18
40 Gy (8Gy x 5 fractions)	1	2
40.05 Gy (2.67Gy x 15 fractions)	2	3
45 Gy (3Gy x 15 fractions)	1	2
48 Gy (4Gy x 12 fractions)	1	2
55 Gy (2.75Gy x 20 fractions)	1	2
56 Gy (4Gy x 14 fractions)	1	2
60 Gy (4Gy x 15 fractions)	2	3
64 Gy (2Gy x 32 fractions)	1	2
**Other radiation modality (N = 6)**		
30 Gy (3Gy x 10 fractions)	6	100

Abbreviations: RT, radiotherapy; SBRT, stereotactic body radiotherapy; SRS, stereotactic radiosurgery; SRT, stereotactic radiotherapy.

Supplementary Table S3: Best local response of each radiated oligoprogressive lesion.

	**Total number of oligoprogressive lesions (N = 139)**	
	**N**	**%**
**Response status**		
Complete response	22	16%
Partial response	43	31%
Stable disease	49	35%
Progressive disease	14	10%
Unevaluable	11	8%

Supplementary Table S4: Best local response of each radiated oligoprogressive lesion among patients receiving stereotactic, non-stereotactic and other radiation modalities.

	Total number of oligoprogressive lesions N (%)				
	**CR**	**PR**	**SD**	**PD**	**UE**
**SBRT / hypofractionated RT (N = 28)**	**3 (11%)**	**10 (36%)**	**10 (36%)**	**2 (7%)**	**3 (11%)**
Adrenal gland (N = 6)	2 (33%)	1 (17%)	3 (50%)	0 (0%)	0 (0%)
Bone (N = 2)	0 (0%)	1 (50%)	0 (0%)	1 (50%)	0 (0%)
Liver (N = 1)	0 (0%)	0 (0%)	1 (100%)	0 (0%)	0 (0%)
Lung (N = 15)	1 (7%)	7 (47%)	4 (27%)	0 (0%)	3 (20%)
Lymph node (N = 2)	0 (0%)	1 (50%)	0 (0%)	1 (50%)	0 (0%)
Spine (N = 2)	0 (100%)	0 (0%)	2 (100%)	0 (0%)	0 (0%)
**SRS/SRT, including hypofractionated regimens (N = 43)**	**12 (28%)**	**16 (37%)**	**6 (14%)**	**6 (14%)**	**3 (7%)**
Brain (N = 43)	12 (28%)	16 (37%)	6 (14%)	6 (14%)	3 (7%)
**Non-stereotactic RT (N = 62)**	**7 (11%)**	**17 (27%)**	**29 (47%)**	**6 (10%)**	**3 (5%)**
Adrenal gland (N = 1)	0 (0%)	0 (0%)	0 (0%)	**1 (100%)**	0 (0%)
Bone (N = 9)	0 (0%)	4 (44%)	2 (22%)	2 (22%)	1 (11%)
Brain (N = 1)	0 (0%)	0 (0%)	0 (0%)	0 (0%)	1 (100%)
Infraspinatus and erector spinae muscles (N = 2)	0 (0%)	0 (0%)	2 (100%)	0 (0%)	0 (0%)
Lung (N = 22)	0 (0%)	10 (45%)	11 (50%)	1 (5%)	0 (7%)
Lymph node (N = 15)	5 (33%)	3 (20%)	6 (40%)	0 (0%)	1 (7%)
Pancreas (N = 1)	0 (0%)	0 (0%)	1 (100%)	0 (0%)	0 (0%)
Spine (N = 9)	0 (0%)	0 (0%)	7 (57%)	2 (29%)	0 (14%)
Supraclavicular mass (N = 1)	1 (100%)	0 (0%)	0 (0%)	0 (0%)	0 (0%)
Upper arm lesion (N = 1)	1 (100%)	0 (0%)	0 (0%)	0 (0%)	0 (0%)
**Other radiation modality recipients (N = 6)**	**0 (0%)**	**0 (0%)**	**4 (67%)**	**0 (0%)**	**2 (33%)**
Brain (N = 6)	0 (0%)	0 (0%)	4 (67%)	0 (0%)	2 (33%)

Abbreviations: CR, complete response; PD, progressive disease; PR, partial response; RT, radiotherapy; SBRT, stereotactic body radiotherapy; SD, stable disease; SRS, stereotactic radiosurgery; SRT, stereotactic radiotherapy; UE, unevaluable.

Supplementary Table S5: Severe treatment related adverse events attributed to immune checkpoint inhibitors.

	**Total number of study patients (N = 103)**	
	**N**	**%**
Pneumonitis	5	5%
Acute kidney injury	1	1%
Nephritis	1	1%
Diarrhoea	1	1%
Pancreatitis	1	1%
Xerostomia	1	1%

Supplementary Table S6: Patterns of first failure following radiation to oligoprogressive lesions among treated patients (N = 64).

	**Total number of patients (N = 64)**	
	**N**	**%**
**Nature of disease progression**		
Patients with progression of pre-existing lesions which were previously responsive or stable on last ICI	17	27%
Patients developing new lesions	16	25%
Patients developing new lesions and having progression of pre-existing lesions which were previously responsive or stable on last ICI	13	20%
Patients with progression of radiated oligoprogressive lesions	10	17%
Patients with progression of pre-existing lesions which were previously responsive or stable on last ICI and radiated oligoprogressive lesions	3	5%
Patients developing new lesions and having progression of radiated oligoprogressive lesions	3	5%
Patient developing new lesions, having progression of pre-existing lesions as well as radiated oligoprogressive lesion	2	3%

Abbreviation: ICI, immune checkpoint inhibitor.
